# AZ304, a novel dual BRAF inhibitor, exerts anti-tumour effects in colorectal cancer independently of BRAF genetic status

**DOI:** 10.1038/s41416-018-0086-x

**Published:** 2018-05-14

**Authors:** Rui Ma, Ling Xu, Xiujuan Qu, Xiaofang Che, Ye Zhang, Yibo Fan, Ce Li, Tianshu Guo, Kezuo Hou, Xuejun Hu, Lisa Drew, Minhui Shen, Tony Cheung, Yunpeng Liu

**Affiliations:** 1 Department of Medical Oncology, The First Hospital of China Medical University, 110001 Shenyang, China; 2 Key Laboratory of Anticancer Drugs and Biotherapy of Liaoning Province, The First Hospital of China Medical University, 110001 Shenyang, China; 3 Department of Respiratory Medicine, The First Hospital of China Medical University, 110001 Shenyang, China; 4 Oncology iMED, AstraZeneca R&D Boston, 35 Gatehouse Drive, Waltham, MA 02451 USA

**Keywords:** Cancer, Cancer

## Abstract

**Background:**

BRAF mutation is associated with poor clinical outcome of patients with malignant tumours, and mediates resistance to chemotherapy and targeted therapy. This study aimed to determine whether V600E mutant and wild type BRAF colorectal cancers exhibit distinct sensitivities to the dual BRAF inhibitor AZ304.

**Methods:**

Kinase activity was assessed by the AlphaScreen assay. Then, MTT assay, EdU assay, colony-formation assay and Western blot were performed to evaluate the anti-tumour effects of AZ304 in vitro. In vivo efficacy was investigated by xenograft analysis and immunohistochemistry.

**Results:**

AZ304 exerted potent inhibitory effects on both wild type and V600E mutant forms of the serine/threonine-protein kinase BRAF, with IC_50_ values of 79 nM and 38 nM, respectively. By suppressing ERK phosphorylation, AZ304 effectively inhibited a panel of human cancer cell lines with different BRAF and RAS genetic statuses. In selected colorectal cancer cell lines, AZ304 significantly inhibited cell growth in vitro and in vivo, regardless of BRAF genetic status. In addition, the EGFR inhibitor Cetuximab enhanced the potency of AZ304 independently of BRAF mutational status.

**Conclusions:**

The BRAF inhibitor AZ304 has broad spectrum antitumour activity, which is significantly enhanced by combination with Cetuximab in colorectal cancers in vitro and in vivo.

## Introduction

The oncogenic BRAF mutations are found in approximately 8% of all human cancers, including 40–70% of melanoma, 36–53% of thyroid, and 5–22% of colorectal cancer (CRC) cases. In addition, BRAF mutations are also present in non-small cell lung cancer, ovarian cancer, gliomas, leukaemia and other malignancies.^1^ The serine/threonine-protein kinase BRAF belongs to the RAF family of kinases, which also include ARAF and CRAF.^2^ As the most common mutation, BRAF V600E mutation causes constitutive activation of downstream signalling through the mitogen-activated protein kinase (MAPK) pathway.^2^ BRAF mutation is currently considered one of the poor prognostic markers in a series of cancers.^1^ In CRC, BRAF mutations are also associated with significantly low response rate with Cetuximab used as a single agent or in combination with chemotherapy for tumours containing wild type RAS.^3–5^

In the past few years selective BRAF inhibitors, such as vemurafenib and dabrafenib have been approved by FDA and EMEA for the treatment of metastatic melanomas harbouring V600E mutant BRAF.^6, 7^ Although BRAF inhibitors produce clinical responses, e.g., improvement of progression free survival and overall survival in patients with mutant BRAF melanoma, the associated effects are short-lived. Different from V600E mutant BRAF cells, many existing BRAF inhibitors paradoxically activate RAF and ERK signalling via a RAS dependent mechanism in wild type BRAF cells.^8^ Furthermore, malignant tumours with V600E mutant BRAF do not respond uniformly to BRAF-targeted therapy.^9^ The majority of colorectal cancer patients harbouring V600E mutant BRAF display inherent resistance to vemurafenib. The overall response rate was only 5% in a clinical trial.^10^ Acquired and intrinsic resistance to BRAF inhibitors likely due to multiple mechanisms, including MAPK pathway activation via CRAF, EGFR/MAPK pathway reactivation, BRAFV600E amplification, SRC/STAT3 pathway upregulation, mutation of NRAS and MEK1, PI3K/AKT pathway activation, and others.^10–17^ Hence, it is necessary to identify and develop more potent BRAF inhibitors.

Our results demonstrated that AZ304, a dual BRAF kinase inhibitor, exerts potent anti-tumour effects on both wild type and mutant BRAF cancer lines. Moreover, combining AZ304 and the anti-EGFR monoclonal antibody Cetuximab resulted in significantly improved anti-tumour activity in colorectal cancer cells both in vitro and in vivo, independently of BRAF mutation status.

## Methods

### Cell culture

Human melanoma cell lines A375 and SK-MEL-31were obtained from ATCC (Manassas, VA). The cells grown in DMEM medium supplemented with 2 mM L-glutamine and 10% foetal bovine serum. Cells was maintained under 37 °C humidified atmosphere containing 5% CO_2_. Human CRC cell lines RKO, HT-29 and Caco-2 were obtained from the Type Culture Collection of the Chinese Academy of Sciences (Shanghai, China). DiFi cell line was purchased from Shanghai Bai Li biological technology Co., Ltd (Shanghai, China). DiFi cells were cultured in MEM and RKO, HT-29 and Caco-2 were grown in RPMI 1640 medium (Gibco, Gaithersburg, MD, USA). All medium contained 10% heat-inactivated foetal bovine serum (FBS), penicillin (100 U/ml), and streptomycin (100 μg/ml) in an atmosphere of 95% air and 5% CO_2_ at 37 °C. Cells were routinely passed every 2–3 days and all cells maintained in culture for a maximum 8 weeks. For all other cells, their information was described in ref.^18^

### Reagents and antibodies

AZ304 and AZ138 were synthesised at AstraZeneca plc (London, UK). Cetuximab was obtained from Merck KgaA (Darmstadt, Germany). EGF was obtained from Pepro Tech (Rocky, USA), Antibodies of p-EGFR (Tyr1068) (2234 S), EGFR (2646 S), BRAF (4933 S), p-ERK1/2 (Thr202/Tyr204) (4370 S), p-p38 (Thr180/Tyr182) (9216 S), p38 (9218 S), p-AKT (Ser473) (4060 S), AKT (9272 S), p-mTOR (Ser2448) (2971 S), SRC (2109 S), p-SRC (Y416) (6943 S), STAT3 (4904 S), p-STAT3 (Tyr705) (9145 S), Caspase-9 (9508 S), Caspase-3 (9662 S) and PARP (9542 L) were obtained from Cell Signalling Technology (Danvers, MA, USA). Antibody of ERK (sc-514302), Actin (sc-1616-R), mTOR (sc-1550-R), goat anti-rabbit IgG and goat anti-mouse IgG were purchased from Santa Cruz Biotechnology (Santa Cruz, CA, USA). Anti-Ki67 antibody was purchased from Fuzhou Maixin Biological Technology (Fujian, China).

### In-vitro enzymatic assay

For RAF kinases, detailed assay information was described in ref.^18^ Briefly, the kinase activity of BRAF WT, BRAF V600E, or CRAF was measured using an AlphaScreen assay (Perkin Elmer, MA) monitoring MEK1/2 phosphorylation at Ser217/221. For CDK2 and CDK4 kinases, activity was also measured by AlphaScreen, monitoring phosphorylation of biotin Rb peptide at Ser780. Similarly, MAP3K7, CSF1R and JAK2 kinase activity was measured by phosphorylation of their biotinylated substrates MKK6 kinase dead protein at Ser271/Thr275, or tyrosine phosphorylation of pEY or Tyk2 Tyr1054/1055 peptides, respectively. CSK, IGF1R, EGFR, FGFR1 and SRC kinase activity was measured using a sandwich ELISA detecting phosphorylated poly EAY peptide with a HRP conjugated phosphotyrosine antibody and TMB substrate, while p38 kinase activity was measured by monitoring phosphorylation of MBP protein with radiolabeled ^33^P-ATP in a filter binding format. All assays were screened under respective ATP Km conditions and inhibitor IC50s were derived from either 5 (RAF kinases, CDK2, CDK4, MAP3K7, JAK2), 10 (CSK, IGF1R, EGFR, FGFR1, SRC) or 11 (p38, CSF1R) point compound dose response.

### Cell proliferation assay

The proliferation assays against a cell panel were performed as described in ref.^18^ Briefly, the cells were treated with DMSO or multiple concentrations of AZ304 for 3 days. The cell growth was determined using the CellTiter 96 Aqueous One Cell Proliferation Assay (Promega, Madison, WI). Percentage of net growth at day 3 (100%) relative to day 0 (0%) was calculated and the concentration of compound required to inhibit growth by 50% (GI_50_) determined. The assays were done in triplicate across different plates.

The proliferation assays in selected colorectal cancer cell lines were measured using a 3-(4,5-dimethyl thiazol-2-yl)-2,5-diphenyl tetrazolium bromide (MTT) assay. First of all, cultured cells were seeded into 96-well plates (2000–5000 cells per well). After incubation for 24 h, the cells were pretreated with DMSO or AZ304 for 1 h. Then the indicated doses of Cetuximab were added. Cells were incubated for a further 48 or 72 h. For EGF stimulating assay, cells were incubated in reduced serum medium overnight and then treated with AZ304 or AZ304 + EGF (20 ng/ml) for 72 h. Twenty microliter of MTT solution (5 mg/ml) was added to each well followed by 4 h incubation at 37 °C. The cell culture medium was removed and the cells were lysed in 200 μl dimethylsulphoxide (DMSO) and the results were measured using a microplate reader (Model 550, Bio-Rad Laboratories, Hercules, CA, USA).

### The EdU incorporation assay

Selected colorectal cancer cells were seeded into 96-well plates (2000–5000 cells per well). After incubation for 24 h, the cells were pretreated with DMSO or AZ304 for 24 h. Then 50 μM of 5-ethynyl-2′-deoxyuridine (EdU, Ribobio, Guangzhou, China) was added to each well and the cells were incubated at 37 °C for 2 h before being fixed with 4% formaldehyde for 30 min and incubated with 2 mg/ml glycine for 5 min. After being washed with PBS for five times, the cells were reacted with 100 μL of 1 × Apollo reaction cocktail for 30 min. Afterwards, the 1 × Hoechst 33342 (5 μg/mL) was used to stain the nuclei.

### Colony-forming assay

Colorectal cells were plated at 300 cells (RKO, HT-29), 500 cells (Caco-2, DiFi*)* into each well of 12-well plates in the medium containing 10% FBS. Cells were incubated at 37 °C in 5% CO_2_ for overnight. Then cells were treated with DMSO or 0.5 μM AZ304 and/or 10 μg/ml Cetuximab. For EGF stimulating assay, cells were pre-incubated for 24 h in reduced serum medium and then treated with DMSO, EGF, AZ304 and AZ304 + EGF. After 14 days, cells were stained by Wright–Giemsa. Finally, the number of colonies was counted by light microscopy.

### A375 p-ERK cellular assay

A375 cells were seeded into 96-well micro plates (Costar, Corning, and Lowell, MA) at 2 × 10^5^ cells/well in phenol red free DMEM (Invitrogen, Carlsbad, CA) supplemented with 10% foetal bovine serum. After 48 h, the cells were treated with DMSO or multiple concentrations of AZ304 and then returned to the incubator for 75 min. Medium were then aspirated and cells were fixed with a 6% formaldehyde solution for 20 min at room temperature. Cells were washed once with PBS containing 0.05% Triton X-100 (PBST) and 0.6% hydrogen peroxide added for 20 min at room temperature. After washing again in PBST, cells were blocked with 10% FBS/PBST solution for 1 h at room temperature. After washing, p-ERK monoclonal antibody was added and the plates were placed at 4 °C for overnight. Plates were then washed in PBST, incubated with goat anti-mouse HRP-conjugated secondary antibody (Cell Signalling Technology, Danvers, MA) for 2 h at room temperature, washed in PBST, ABTS solution (Sigma, St. Louis, MO) added and plates incubated for 2 h at 30 °C. Quantification of signal was determined at OD_405_ using a SpectraMax plate reader (Molecular Devices, Sunnyvale, CA). All assays were done in duplicate across different plates. EC_50_ was calculated using XLift.

### Western blot analysis

For western-blotting in A375, A549 and MC-F7 cell lines, the detailed method was described in ref.^18^ The cells were collected after 75 min treatment with DMSO or at 0.1, 1, 10 and 100-fold of AZ304 IC50 value in A375 p-ERK cellular assay. For western-blot in selected CRC cell lines, the cells were collected after treatment for indicated time and washed twice with cold PBS. Then cells were lysed in 1% Triton lysis buffer (50 mmol/L Tris-HCl, pH 7.4, 10 mmol/L EDTA, 100 mmol/L NaF, 150 mmol/L NaCl, 1% Triton X-100, 1 mmol/L PMSF 1 mmol/L Na3VO4 and 2 μg/mL aprotinin). After sonication and centrifugation, samples were quantified with the Lowry method. Next, all the protein samples were boiled at 95 °C for 5 min with 3× sampling buffer. After that, all the lysate samples were resolved by SDS polyacrylamide gel electrophoresis (SDS-PAGE) and followed by western blotting which means electronically transferred to nitrocellulose membranes. After blocking with 5% skim milk in TBST buffer (10 mM Tris-HCl pH 7.4, 150 mM NaCl, 0.1% Tween 20) at room temperature for 1 h, the blots were incubated with indicated antibodies shaking for 2 h, then overnight at 4 °C. Then the blots were washed four times with TBST buffer, and then incubated with secondary antibodies for 30 min at room temperature. After washing for another four times, the protein bands were detected with enhanced chemiluminescence reagent (SuperSignal Western Pico Chemiluminescent Substrate; Pierce, Rockford, IL, USA) and visualised with the Electrophoresis Gel Imaging Analysis System (DNR Bio-Imaging Systems, Jerusalem, Israel).

### Xenograft studies

All in vivo experiments were performed in accordance with Institutional Review Board of China Medical University guidelines. Female 4–6 weeks old athymic BALB/c nude mice were purchased from Shanghai SLAC Laboratory Animal Centre (Shanghai, China). RKO/Caco-2 cells (1 × 10^7^) in 200 μl PBS were injected subcutaneously into the right scapular region of mice. After the average tumour size reached 150 – 200 mm^3^, animals were randomly divided into 4 groups, each containing three mice and were treated with vehicle only (CON) which orally received 0.5% HPMC and injected with 0.9% saline, AZ304 only (AZ304 dissolved in 0.5% HPMC, 10 mg/kg by oral gavage twice daily), Cetuximab only (40 mg/kg by intraperitoneal injection twice per week), or their combination (A+C) for 10 days. Tumours were measured with a caliper every 2 days, so did body weights. Tumour volume was calculated using the formula V = 1/2 (width^2^ × length). Mice were terminated by CO_2_ inhalation when the tumour diameters reached 1.5 cm, according to the protocol filed with the Guidance of Institutional Animal Care and Use Committee of China Medical University.

### Immunohistochemistry

Tumours were formalin-fixed, paraffin-embedded and prepared as described in our previous study for staining with haematoxylin and eosin.^19^ The immunohistochemical antibodies Ki67, p-ERK, p-EGFR and p-AKT have been described already. The staining was evaluated by scanning the entire tissue specimen under low magnification (×10) and confirmed under high magnification (×20 and ×40). The protein expression was visualised and classified based on the percentage of positive cells and the intensity of staining. From each section, five visual fields were randomly selected. The degree of protein expression was based on the percentage of positive cells and the intensity of staining. Staining intensity was scored as 0 (no staining), 1 (low staining), 2 (intermediate staining), and 3 (high staining). For the staining area, ≤5%, 5–25%, 26–50, 51–75% >75% were recorded as 0, 1, 2, 3 and 4 points, respectively. Histological score = staining intensity × staining area. A score 0 was classified as negative (−), 1–4 points as weakly positive (+), 6–12 points as a strong positive (++). Final scores were assigned by two independent pathologists.

### Statistical analysis

All the presented data were verified by three separate experiments, and are expressed as the means ± standard deviation (SD). Differences between groups were calculated by Student’s two-tailed *t*-test. All analyses were calculated using SPSS 20.0 software. *P* < 0.01 and *P* < 0.05 were considered statistically significant. IC50, EC50 and GI50 values were determined with GraphPad Prism 6 software.

## Results

### AZ304 is a potent dual BRAF inhibitor of both wild type and V600E mutant BRAF kinases

**Fig. 1:**
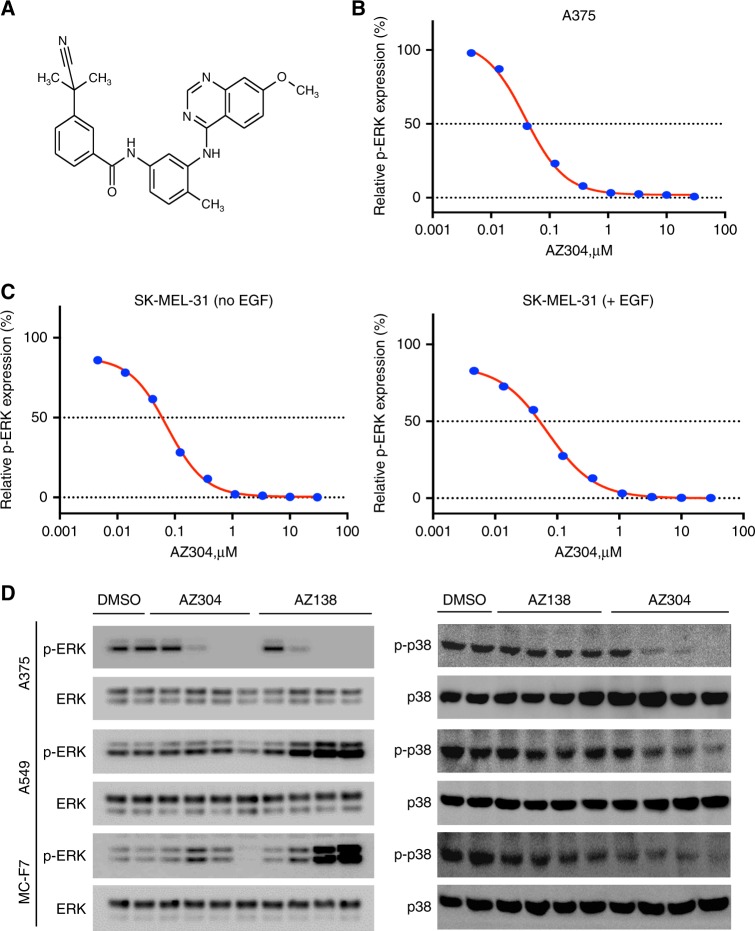
AZ304 is a potent inhibitor of both wild type and mutant BRAF kinase. **a** AZ304 chemical structure. **b** A375 p-ERK cellular assay: AZ304 concentration dependently reduced p-ERK levels in A375 cells. Cells were incubated with DMSO or AZ304 for 75 min and p-ERK levels were determined using a p-ERK antibody based ELISA. A representative dose response curve is shown. **c** SK-MEL-31 p-ERK cellular assay: AZ304 concentration dependently reduced p-ERK levels in SK-MEL-31 cells. Cells were incubated with DMSO or AZ304 (±EGF) for 75 min and p-ERK levels were determined using a p-ERK antibody based ELISA. A representative dose response curve is shown. **d** In vitro p-ERK and p-P38 evaluation in cell lines containing wild type BRAF or V600E mutant BRAF: Cells were collected following 75 min treatment at 0.1, 1, 10 and 100 fold of A375 p-ERK EC50 for each compound. (A375: BRAF V600E, A549: RAS MT; MC-F7: BRAF/RAS WT)

AZ304 is a synthetic inhibitor designed to interact with the ATP-binding site of wild type and V600E mutant BRAF (Fig. 1a). It showed potent inhibitory activities to the kinase domains of wild type BRAF, V600E mutant BRAF and wild type CRAF in vitro, with IC50 values of 79 nM, 38 nM and 68 nM, respectively (Table 1). Further profiling of AZ304 activity against other selected kinases revealed its inhibition of two other kinases, including p38, and CSF1R (Table 1). Consistent with BRAF kinase inhibition in vitro, AZ304 potently reduced ERK phosphorylation (p-ERK), with a mean EC50 of 65 nM in the V600E mutant BRAF containing melanoma cell line A375 (Fig. 1b); an EC50 of 60 nM was obtained for the wild type BRAF melanoma cell line SK-MEL-31(Fig. 1c). Moreover, EGF stimulation did not effectively increase p-ERK levels of SK-MEL-31, and the EC50 was 52 nM (Fig. 1c). As a result, EC50 values with and without EGF stimulation were comparable. A concentration dependent reduction of p-ERK was also observed in the wild type BRAF cell lines A549 and MC-F7 treated with AZ304. However, AZ138, a V600E mutant BRAF specific inhibitor, paradoxically activated p-ERK in wild type BRAF cell lines (Fig. 1d). Meanwhile, AZ304 potently inhibited p-p38 in cell lines of both BRAF genetic statuses, but not AZ138 (Fig. 1d). Furthermore, AZ304 markedly inhibited cell proliferation in mutant BRAF cancer cell lines, and effectively reduced cell growth in selected cell lines harbouring wild type BRAF/RAS or mutant RAS. The GI50 values ranged from 0.08–7.72 μM in mutant BRAF cell lines, 0.43–11.7 μM in wild type BRAF/RAS cell lines, and 0.9–16.66 μM in mutant RAS cell lines (Supplementary Table 1). AZ304 exhibited anti-proliferative effects on multiple cancer types, including melanoma, colorectal cancer, leukaemia, ovarian cancer, lung cancer, and pancreatic cancer, independently of BRAF genetic status. AZ304 potently inhibits the kinase activity of human RAF enzymes. In this study, AZ304 inhibited wild type BRAF, V600E mutant BRAF, and CRAF, as well as p38 and CSF1R, at sub 100 nM potencies, with selectivity towards other kinases shown.

**Table 1:** Enzyme IC50 values of AZ304 against a panel of kinases

Kinase	IC50 (nM)
BRAFV600E	38
BRAF	79
CRAF	68
p38	6
CSF1R	35
MAP3K7	6400
CSK	7050
IGF1R, EGFR, FGFR, CDK2, CDK4, JAK2, SRC	>10000

### AZ304 inhibits cell proliferation and downregulates ERK phosphorylation in both V600E mutant and wild type BRAF CRC cell lines, whereas Cetuximab is only effective in wild type BRAF cells

**Fig. 2:**
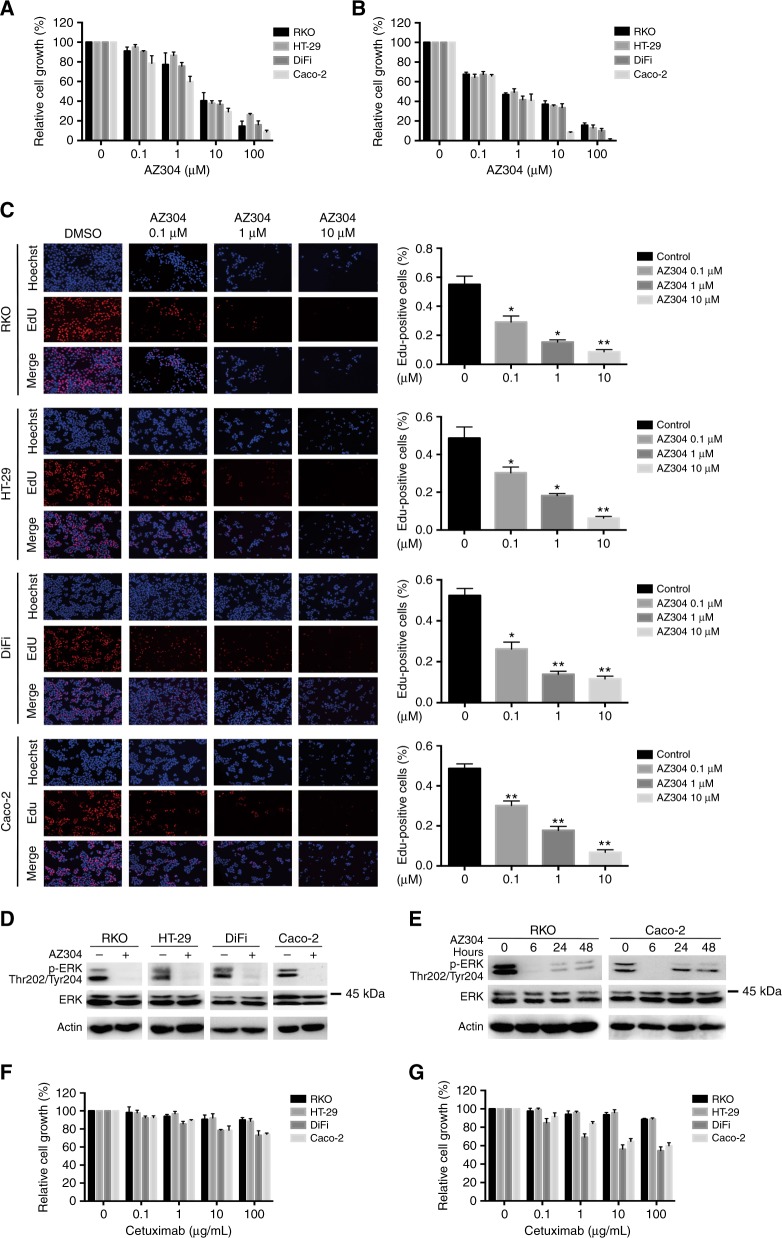
Anti-proliferative activity of AZ304 and Cetuximab in CRC cell lines with different BRAF mutation status. Two V600E mutant BRAF cell lines (RKO, HT-29) and two wild-type BRAF cell lines (DiFi, Caco-2) were treated with DMSO or increasing concentrations of AZ304 (0, 0.1, 1, 10, 100 μM), for 48 h (**a**) and 72 h (**b**). Viable cells were determined by MTT assay. **c** EdU incorporation assay. Four cells lines from (**a**) were treated with DMSO or indicated AZ304 for 24 h, followed by incubation with EdU and Hoechst in sequence. Hoechst 33342 (blue) and EdU (red) represent cell nuclei and nuclei of proliferative cells, respectively. The percentages of the EdU-positive cells are presented (right). Student’s *t*-tests were used for statistical analyses. Data are plotted as mean ± SD. *P < 0.05 vs. control; **P < 0.01 vs. control. **d** These four cells lines from (**a**) were treated with DMSO or 2 uM AZ304 for 6 h. Expression levels of phosphorylation ERK were analysed by Western blot. **e** BRAF mutant cell line RKO, and BRAF wild type cell line Caco-2 were treated with DMSO or 2 uM AZ304 for the indicated times. Expression levels of phosphorylation ERK were analysed by Western blot. The four cell lines were treated with increasing concentrations of Cetuximab (0, 0.1, 1, 10, 100 μg/ml) for 48 h (**f**) and 72 h (**g**). Cell viability was determined by MTT assay

To further assess whether the BRAF mutation status affects CRC cell viability after AZ304 treatment, anti-proliferative effects of AZ304 were determined against four CRC cell lines, including two V600E mutant (RKO and HT-29) and two wild type (DiFi and Caco-2) BRAF cell lines, respectively. Treatment with AZ304 resulted in decreased cell viability in all four CRC cell lines, in a time and concentration dependent manner (Fig. 2a, b). AZ304 concentrations that inhibited 50% cell growth (GI_50_) according to BRAF genetic status are shown in Table 2. Next, the anti-proliferative activities of AZ304 against four CRC cell lines were examined by the EdU assay. As shown in Fig. 2c, AZ304 suppressed DNA replication in CRC cells at all concentrations. Consistent with cell proliferation assay results, AZ304 reduced p-ERK levels in all four CRC cell lines (Fig. 2d). However, p-ERK levels began to increase after 24 h of treatment in CRC cells, independently of BRAF mutation status (Fig. 2e). Subsequently, cells were treated with the indicated concentrations of Cetuximab. As shown in Fig. 2f, g, only wild type BRAF cell lines were sensitive to Cetuximab, while mutant BRAF cell lines were relatively insensitive or resistant. Cell viability after treatment with 10 μg/ml Cetuximab for 72 h is shown in Table 2. The results demonstrated that AZ304 inhibited cell proliferation in selected CRC cell lines, independently of BRAF mutation status, while the EGFR inhibitor Cetuximab only inhibited CRC cell lines with wild type BRAF.

**Table 2:** GI50 values of AZ304 and Cetuximab in Selected CRC cell lines with containing different BRAF genetic statues

Cell line	GI50 (μM) of AZ304 48 h	GI50 (μM) of AZ304 72 h	Relative cell growth (%) of Cetuximab (10 μg/ml)	BRAF mutation	KRAS mutation
RKO	4.539	0.5032	93.60 ± 2.47	MT (V600E)	WT
HT-29	3.896	0.3887	95.90 ± 3.20	MT (V600E)	WT
DiFi	4.987	0.6354	56.28 ± 4.71	WT	WT
Caco-2	1.763	0.3772	64.80 ± 2.93	WT	WT

*WT* wildtype, *MT* mutant

### Cetuximab increases the anti-proliferative activity of AZ304 in both V600E mutant and wild type BRAF CRC cell lines

**Fig. 3:**
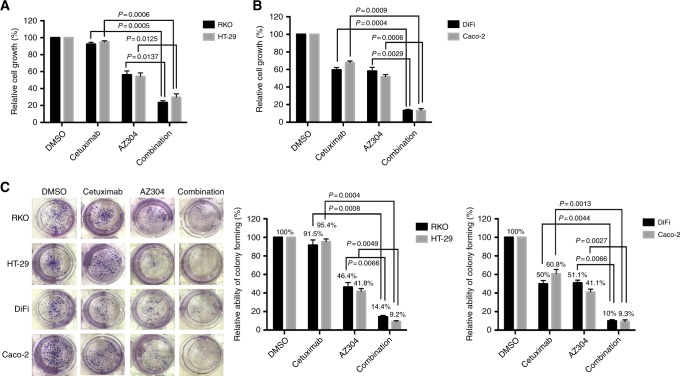
Cetuximab enhances the growth inhibitory effects of AZ304 in all four CRC cell lines. **a**, **b** Two V600E mutant BRAF cell lines (RKO, HT-29) and two wild type BRAF cell lines (DiFi, Caco-2) were exposed to DMSO, 0.5 μMAZ304, 10 μg/ml Cetuximab or the combination of two drugs for 72 h. Cell growth was determined by MTT assay. Student’s *t*-tests were used for statistical analyses. Data are plotted as mean ± SD. *P* values were labelled in the figures. **c** Cells were treated with DMSO, 0.5 μM AZ304, 10 μg/ml Cetuximab and the combination of two agents for 14 days and a colony forming assay was performed, and the clones were counted. Student’s t-tests were used for statistical analyses. Data are plotted as mean ± SD. *P* values were labelled in the figures

To assess whether Cetuximab potentiated the anti-tumour activity of AZ304, CRC cell lines were treated with AZ304 in combination with Cetuximab. The results obtained with combined treatment with AZ304 and Cetuximab in all four CRC cell lines are shown in Fig. 3a, b. Compared with either agent alone, the Cetuximab and AZ304 combination significantly reduced cell growth in all four CRC cell lines. V600E mutant BRAF CRC cell lines were resistant to Cetuximab; however, Cetuximab combined with AZ304 decreased cell viability in RKO cells (AZ304, 56.20% ± 4.52 vs. 23.48% ± 2.18, *P* = 0.0137) and HT-29 cells (AZ304, 54.26% ± 4.23 vs. 29.73% ± 4.06, *P* = 0.0125). In wild type BRAF cells, although monotherapy inhibited proliferation, the combination further enhanced these effects: DiFi cells (AZ304, 58.13% ± 4.28 vs. 13.32% ± 1.09, *P* = 0.0029; Cetuximab, 59.57% ± 2.68 vs. 13.32% ± 1.09, *P* = 0.0004); Caco-2 cells (AZ304, 51.90% ± 2.21 vs. 13.30% ± 2.18, *P* = 0.0006; Cetuximab, 67.86% ± 1.82 vs. 13.30% ± 2.18, *P* = 0.0009). Consistent with short-term proliferation assays, colony-formation assay of all four cell lines revealed that treatment with AZ304 in combination with Cetuximab produced fewer and smaller colonies compared with the monotherapy groups (Fig. 3c). These results suggested that Cetuximab could increase AZ304-associated inhibition of cell proliferation regardless of BRAF mutation status.

### AZ304 suppresses three cell proliferation pathways, including MAPK/ERK, PI3K/AKT/mTOR, and SRC/STAT3 pathways, effects enhanced by combination with Cetuximab, independently of BRAF mutation status

**Fig. 4:**
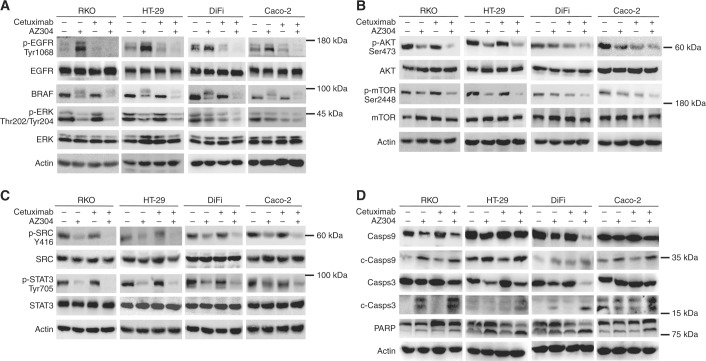
Cetuximab enhance inhibition of cellular targets and apoptosis effect of AZ304 independent of BRAF genetic status. Two mutant BRAF lines (RKO, HT-29) and two wild type BRAF cell lines (DiFi, Caco-2) cell lines were exposed to DMSO, 2 μMAZ304, 10 μg/ml Cetuximab or the combination of two drugs for specific time (**a**, **b**, **c** 36 h, **d** 48 h). **a** Western blot analysis was used to detect the phosphorylation of EGFR, BRAF, ERK. **b** Western blot analysis was used to detect the phosphorylation of AKT, mTOR. **c** Western blot analysis was used to detect the phosphorylation of SRC, STAT3. **d** Western blot analysis was used to detect the induction of cleaved caspases and cleaved PARP

To explore the mechanism by which AZ304 and its combination with Cetuximab inhibit cancer cell proliferation, EGFR and its downstream signalling pathways were evaluated after treatment with AZ304 and/or Cetuximab of four CRC cell lines. Initially, we analysed the most common proliferation pathways downstream of EGFR, including mitogen-activated protein kinase/extracellular regulated protein kinases (MAPK/ERK) and phosphoinosmde-3-kinase/AKT/ mammalian target of rapamycin (PI3K/AKT/mTOR) pathways, separately. Treatment with AZ304 alone decreased BRAF, p-ERK, p-AKT and p-mTOR levels in both BRAF V600E mutant and BRAF wild type cells (Fig. 4a, b). As previous reports showed that feedback activation of EGFR is elicited by BRAF inhibitor, we observed that EGFR phosphorylation (p-EGFR) was increased by AZ304 in both mutant and wild type BRAF cells. Accordingly, combination with Cetuximab showed a significant down-regulation of p-EGFR. Meanwhile, the combination achieved sustained inhibition of p-ERK in both mutant and wild type BRAF cell lines (Fig. 4a, b). See the supplementary Fig. 1 for quantity and histograms for the phosphorylation markers. Consequently, in all four CRC cell lines tested, combination of AZ304 and Cetuximab caused a more potent inhibition of BRAF, ERK, AKT and mTOR signalling pathways, compared with single agents, providing a rationale for the observed synergy in anti-tumour effects.

It was reported RAF inhibitors with anti-SRC activity show improved anti-proliferative activities in BRAF-mutant melanoma cells.^20^ To assess whether AZ304 plays a role in blocking SRC tyrosine kinase activation, the effects of single and combined treatments on SRC and STAT3 phosphorylation were examined (Fig. 4c). Following exposure to AZ304, the four CRC cell lines all exhibited relatively reduced expression levels of phosphorylated SRC and STAT3. Furthermore, irrespective of BRAF mutation status, AZ304 and Cetuximab combination showed an even more pronounced inhibition of both SRC and STAT3 protein phosphorylation levels (Fig. 4c).

To determine whether the above treatments could induce apoptosis in CRC cell lines, activation of caspases and PARP was evaluated. Treatment with AZ304 alone resulted in overtly increased levels of cleaved caspase-9, caspase-3 and PARP in all the four cell lines tested. After combined treatment of AZ304 and Cetuximab, the expression levels of cleaved biomarkers were substantially increased in all four cell lines, which could contribute to commitment to apoptosis (Fig. 4d). Taken together, these findings indicated that AZ304 inhibited cell proliferation in both BRAF mutant and wild type CRC cells by suppressing three downstream pathways of EGFR and inducing apoptosis. In addition, the EGFR inhibitor Cetuximab further enhanced the effects of AZ304 on these pathways.

### AZ304 retains inhibitory activity against both V600E mutant and wild type BRAF CRC cell lines in the presence of the EGFR ligand EGF

**Fig. 5:**
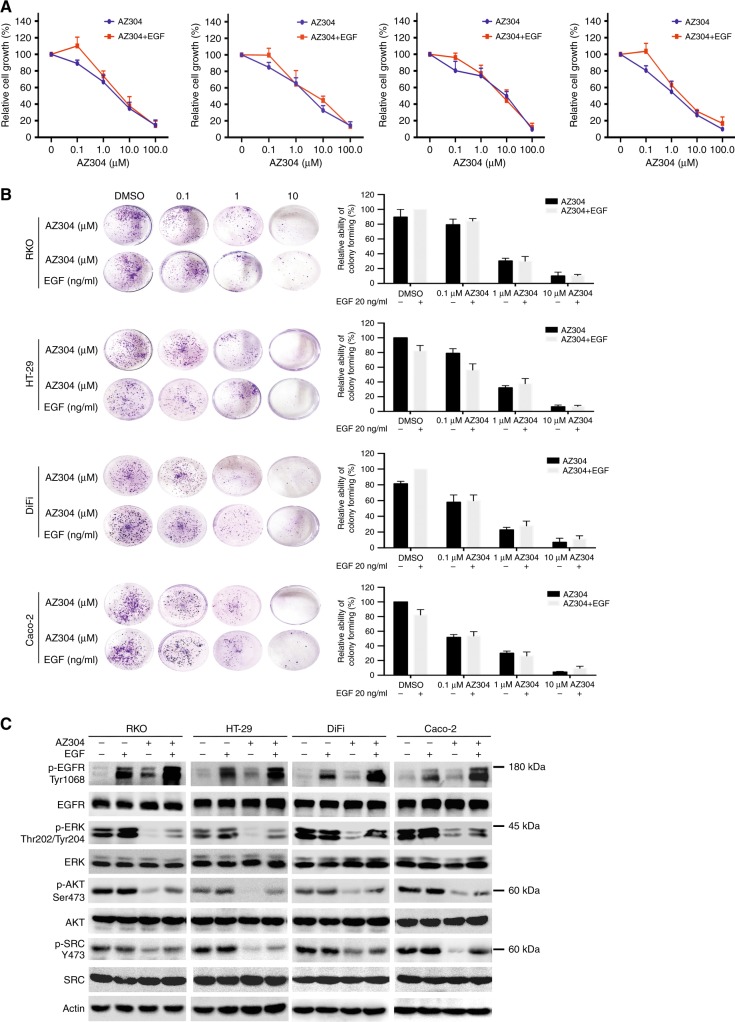
Anti-proliferative activity of AZ304 cannot be retained by EGF in CRC cell lines with different BRAF mutation status. **a** Two V600E mutant BRAF cell lines (RKO, HT-29) and two wild type BRAF cell lines (DiFi, Caco-2) were serum starved overnight and then treated with the indicated AZ304 or AZ304 + EGF for 72 h. Viable cells were determined by MTT assay. **b** These four cells lines from (**a**) were serum starved overnight and then treated with DMSO, EGF, indicated AZ304, and AZ304 + EGF for 14 days and a colony forming assay was performed, and the clones were counted. Student’s t-tests were used for statistical analyses. Data are plotted as mean ± SD. **c** These four cells lines from (**a**) were serum starved overnight and then treated with DMSO, 20 ng/ml EGF, 2 μMAZ304, and AZ304 + EGF for 36 h. Then Western blot analysis was used to detect the phosphorylation of EGFR, ERK, AKT, SRC

To assess whether ligand induced activation of EGFR could affect the anti-proliferative effects of AZ304, CRC cell lines were treated with AZ304 alone or in combination with EGF. Compared with AZ304 alone, there was no significant reduction of anti-proliferative effects after combined treatment with EGF (Fig. 5a). Next, to determine whether the inhibitory effects of AZ304 on cancer cells could be rescued by EGF in long-term proliferation assays, the colony formation assay was implemented. The results showed that the colonies of the 4 CRC cells were markedly inhibited by AZ304 in a dose-dependent manner. In addition, combination with EGF did not significantly impair the anti-proliferative effects of AZ304. (Fig. 5b). Furthermore, whether EGF could alter AZ304 effects on downstream signalling pathways of EGFR in the four CRC cell lines was assessed. Although EGF activated EGFR, AZ304 still blocked downstream signals of EGFR. It was obvious that AZ304 treatment resulted in reduced p-ERK, p-AKT and p-SRC levels, and EGF stimulation in AZ304 treated was able to partially rescue p-ERK, p-AKT and p-SRC inhibition (Fig. 5c). Taken together, the inhibitory effects of AZ304 against both V600E mutant and wild type BRAF CRC cell lines were not overtly altered in the presence of the EGFR ligand EGF.

### Anti-tumour effects of AZ304 with or without Cetuximab on RKO and Caco-2 tumour xenografts

**Fig. 6:**
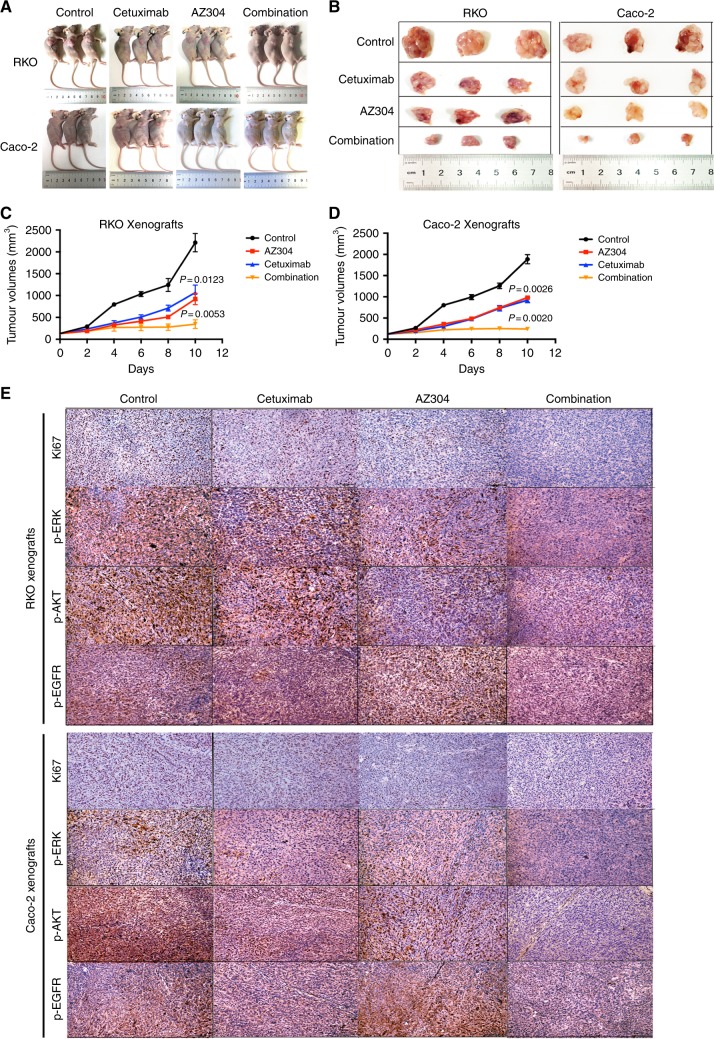
Cetuximab enhances AZ304 anti-tumour activity in RKO and Caco-2 xenograft model. **a**, **b** Photographs of tumours that developed in xenograft transplanted nude mouse tumour models 3 weeks after injection of RKO or Caco-2 cells. Each containing three mice, and were treated with vehicle only(CON), AZ304 only (AZ304, 10 mg/kg by oral gavage twice daily), Cetuximab only (40 mg/kg by intraperitoneal injection twice per week), or their combination for 10 days. In vivo subcutaneous tumour growth curves were shown for RKO (**c**) and Caco-2 (**d**) cells in vivo. Student’s *t*-tests were used for statistical analyses. Data are plotted as mean ± SD. *P* values were labelled in the figures. **e** Tumour tissue from RKO and Caco-2 xenografts treated for 10 days as indicated was evaluated by IHC for Ki67, p-ERK, p-AKT and p-EGFR. Tumours were harvested 4 h after dosing on day 10

Based on the anti-proliferative activity of AZ304 alone and in combination with Cetuximab in CRC cells in vitro, we next tested whether monotherapy and the combination strategy were effective in vivo by using RKO (BRAF mutant colorectal cancer cell) and Caco-2 (BRAF wild type colorectal cancer cell) xenografts in athymic nude mice. The mice were treated with vehicle, AZ304, Cetuximab, or the AZ304 and Cetuximab combination. Sizes and masses of the implanted tumours were measured every two days until study end (Fig. 6a, b). Compared with vehicle-treated controls, treatment with AZ304 or Cetuximab alone resulted in reduced tumour growth in both xenograft models (*P* = 0.0123 in con VS AZ304 for RKO; *P* = 0.0026 in con VS AZ304 for Cacco-2). Furthermore, the AZ304 and Cetuximab combination caused dramatic tumour growth inhibition and even shrinking in the Caco-2 xenograft model (*P* = 0.0020) (Fig. 6c, d). In addition, animal health and body weight were monitored. The results showed that no animals died during the treatment course, and tolerated all treatments without overt signs of toxicity (Supplementary Fig. 2).

To further explore the mechanism of action of different treatments, the tumours were extracted and processed for immunohistochemical staining of Ki67, p-ERK, p-EGFR and p-AKT. As a marker of cell proliferation, Ki67 is expressed by proliferating cells in all phases of the active cell cycle and absent in resting cells. Ki67, p-ERK and p-AKT signals decreased markedly after treatment with AZ304 compared with untreated tumours, as well as the Cetuximab alone group. Moreover, the combination caused more potent inhibitory effects compared with either treatment alone (Fig. 6e). To explore whether p-EGFR might be unregulated by AZ304, p-EGFR levels were assessed in xenograft models generated with both BRAF genetic statuses. Compared with vehicle-treated controls, treatment with AZ304 exhibited significantly higher levels of p-EGFR, corroborating in vitro findings. Furthermore, AZ304 and Cetuximab combination led to improved inhibition of p-EGFR compared with either monotherapy (Fig. 6e and Supplementary Table 2). These results suggested an in vivo anti-tumour efficacy of AZ304 monotherapy and its combination with Cetuximab against CRC cell xenografts without obvious toxicity, independently of BRAF mutation status.

## Discussion

The most common BRAF mutation is the missense mutation of V600E. The BRAF-specific inhibitors vemurafenib and dabrafenib have been approved for the treatment of melanomas harbouring the BRAF V600E mutation, but not BRAF wild type harbouring melanomas.^21^ Moreover, most patients ultimately develop acquired resistance after a relatively short period of remission, and some patients harbouring BRAF mutations present intrinsic resistance to these drugs, e.g., CRC and thyroid cancer patients.^22, 23, 10^ In addition to BRAF mutations, wild type BRAF-mediated ERK activation plays a major role in tumour proliferation of uveal melanoma,^24^ indicating that suppression of both mutant and wild type BRAF can improve antitumour effects. The current clinical BRAF inhibitors vemurafenib and dabrafenib bind all RAF isoforms in kinase assays, but only inhibit proliferation and ERK signalling in BRAF V600E mutant cells.^25^ However, vemurafenib and dabrafenib caused a paradoxical induction of p-ERK and induce proliferation in BRAF wild type cells.^26, 27^ Here, we demonstrated that AZ304, a novel BRAF/BRAF V600E inhibitor, had anti-proliferative effects on both BRAF mutant and wild type tumours in vitro and in vivo. This compound potently inhibited all RAF kinases, including BRAF, BRAF V600E and CRAF, with a feature of kinase selectivity, exhibiting potent inhibition (with IC50 below 100 nM) for p38 and CSF1R. Inhibition of CRAF activity in AZ304 may help decrease the odds of resistance. Then, a concentration dependent reduction of p-ERK was observed in cancer cell lines with both BRAF statuses. It was reported that CSF1R has abnormal expression in various cancer cells, and its activation could upregulate multiple signalling transduction pathways leading to tumour proliferation and metastasis.^28, 29^ By inhibiting CSF1R, AZ304 showed potent anti-tumour activity. As shown above, AZ304 displayed effective anti-proliferative activity independently of BRAF mutation Status.

It is well-known that the selective BRAF inhibitors vemurafenib and dabrafenib reduce cell proliferation by suppressing ERK phosphorylation in tumours with BRAF V600E only.^21^ However, in this study, ERK phosphorylation was inhibited after AZ304 treatment in cell lines and tumour tissues bearing both V600E and wild type BRAF genes. To comprehensively explore the molecular mechanism by which AZ304 prevents cell growth, many other cell proliferation pathways were assessed. It has been suggested that activation of the PI3K/AKT/mTOR and SRC/STAT3 pathway may be involved in resistance to BRAF inhibitors in melanoma and CRC cells.^12, 14^ However, AZ304 inhibited not only PI3K/AKT/mTOR activation, but also that of the SRC/STAT3 pathway in both BRAF mutant and wild type CRC cells, as shown above. Although there were no direct AZ304 effects on AKT and SRC kinases, it was reported that CSF1R promotes tumour cell proliferation by activating the downstream PI3K/AKT/mTOR and SRC/STAT3 pathway.^28, 30, 31^ Therefore, reduction of these phosphorylated proteins may be dependent on the inhibitory effects of AZ304 on cellular epidermal receptors such as CSF1R. In addition, vemurafenib induces apoptosis in BRAF mutant and melanoma cell lines.^32^ In this study, AZ304 also induced caspase-9, caspase-3 and PARP cleavage, both in V600E mutant and wild type BRAF CRC cells. These findings indicated that AZ304 exerts anti-proliferative effects on CRC cells by inhibiting survival signalling pathways and increasing apoptosis, independently of BRAF mutational status. However, the detailed mechanisms by which AZ304 exerts anti-tumour effects still need in-depth investigation.

It is known that BRAF inhibition increases EGFR activity, which is considered one of the mechanisms of CRC resistance to BRAF inhibition,^10, 33^ and combination of BRAF and EGFR inhibitors suppressed tumour growth in preclinical models.^34, 35^ In the 2017 annual meeting of the American Society of Clinical Oncology (ASCO), it was reported that CRC patients with BRAF mutations simultaneously treated with vemurafenib, Cetuximab, and Irinotecan (VIC) show improved progression free survival (PFS).^36^ Additionally, the vemurafenib and Erlotinib combination is also safe and efficient in CRC patients with BRAF mutation.^37^ In this study, AZ304 feedback induced EGFR activation in V600E mutant and wild type BRAF CRC cells. However, the AZ304 and Cetuximab combination reversed EGFR activation associated with AZ304, and resulted in stronger anti-tumour activity in vitro and in vivo, in RAS wild type CRC cells irrespective of BRAF genetic status. Interestingly, we found that Cetuximab exerted stronger tumour growth inhibitory effects on mutant BRAF (RKO) cell lines in vivo than in vitro. In addition to blocking EGFR, Cetuximab also develops anti-tumour effects by provoking ADCC and inhibiting angiogenesis in vivo.^38–40^ Therefore, the anti-proliferative activity of Cetuximab in vivo is stronger than that in vitro. Moreover, the AZ304 and Cetuximab combination showed a more obvious inhibition of the PI3K/AKT/mTOR and SRC/STAT3 pathway in both BRAF wild type and mutant CRC cells. It was previously demonstrated that the effects of other BRAF inhibitors (for example vemurafenib) can be rescued by EGFR and/or HER-3 ligands.^41, 42^ However, in this study, AZ304 retained its inhibitory effects on both V600E mutant and wild type BRAF CRC cell lines in the presence of the EGFR ligand EGF. These findings demonstrated that AZ304 combined with Cetuximab could inhibit the feedback activation of EGFR signalling associated with AZ304, achieving sustained p-ERK inhibition.

In summary, AZ304, a novel dual BRAF inhibitor, exerts potent anti-proliferative effects on selected cancer cells independently of the BRAF genotype. Cetuximab increased sensitivity to AZ304 in RAS wild type CRC cells irrespective of BRAF genetic status. AZ304 may constitute a novel drug for the treatment of BRAF mutant and wild type human cancers, alone and/or in combination with Cetuximab.

## Electronic supplementary material

Supplementary Figure legends

Supplementary table 1

Supplementary table 2

supplymentary fig1

supplymentary fig2

## Availability of data and material

The study was based entirely on previously published data.

## Note

This work is published under the standard licence to publish agreement. After 12 months the work will become freely available.

## References

[1] Rahman MA, Salajegheh A, Smith RA, Lam AK (2014). BRAF inhibitor therapy for melanoma, thyroid and colorectal cancers: development of resistance and further prospects. Curr. Cancer Drug. Targets.

[2] Mercer KE, Pritchard CA (2013). Raf proteins and cancer: B-Raf is identified as a mutational target. Biochim. Biophys. Acta.

[3] Di Nicolantonio F (2008). Wild-type BRAF is required for response to Panitumumab or Cetuximab in metastatic colorectal cancer. J. Clin. Oncol..

[4] De Roock W (2010). Effects of KRAS, BRAF, NRAS and PIK3CA mutations on the efficacy of Cetuximab plus chemotherapy in chemotherapy-refractory metastatic colorectal cancer: a retrospective consortium analysis. Lancet Oncol..

[5] Pietrantonio F (2015). Predictive role of BRAF mutations in patients with advanced colorectal cancer receiving Cetuximab and panitumumab: a meta-analysis. Eur. J. Cancer.

[6] Bollag G (2012). Vemurafenib: the first drug approved for BRAF-mutant cancer. Nat. Rev. Drug. Discov..

[7] Menzies AM, Long GV (2014). Dabrafenib and trametinib, alone and in combination for BRAF-mutant metastatic melanoma. Clin. Cancer Res..

[8] Karoulia Z (2016). An integrated model of RAF inhibitor action predicts inhibitor activity against oncogenic BRAF signaling. Cancer Cell..

[9] Hyman DM (2015). Vemurafenib in Multiple Nonmelanoma Cancers with BRAF V600 Mutations. N. Engl. J. Med..

[10] Corcoran RB (2012). EGFR-mediated reactivation of mapk signaling contributes to insensitivity of BRAF-mutant colorectal cancers to RAF inhibition with Vemurafenib. Cancer Discov..

[11] Ahronian LG (2015). Clinical acquired resistance to RAF inhibitor combinations in BRAF-mutant colorectal cancer through MAPK pathway alterations. Cancer Discov..

[12] Girotti MR (2013). Inhibiting EGF receptor or SRC family kinase signaling overcomes BRAF inhibitor resistance in melanoma. Cancer Discov..

[13] Nazarian R (2010). Melanomas acquire resistance to B-RAF(V600E) inhibition by RTK or N-RAS upregulation. Nature.

[14] Mao M (2013). Resistance to BRAF inhibition in BRAF-mutant colon cancer can be overcome with PI3K inhibition or demethylating agents. Clin. Cancer Res..

[15] Konieczkowski DJ (2014). A melanoma cell state distinction influences sensitivity to MAPK pathway inhibitors. Cancer Discov..

[16] Villanueva J (2010). Acquired resistance to BRAF inhibitors mediated by a RAF kinase switch in melanoma can be overcome by cotargeting MEK and IGF-1R/PI3K. Cancer Cell..

[17] Nagasawa I, Kunimasa K, Tsukahara S, Tomida A (2017). BRAF-mutated cells activate GCN2-mediated integrated stress response as a cytoprotective mechanism in response to vemurafenib. Biochem. Biophys. Res. Commun..

[18] Vasbinder MM (2013). Discovery and optimization of a novel series of potent mutant B-Raf (V600E) selective kinase inhibitors. J. Med. Chem..

[19] Li H (2014). Ubiquitin ligase Cbl-b represses IGF-I-induced epithelial mesenchymal transition via ZEB2 and microRNA-200cregulation in gastric cancer cells. Mol. Cancer.

[20] Girotti MR (2015). Paradox-breaking RAF inhibitors that also target SRC are effective in drug-resistant BRAF mutant melanoma. Cancer Cell..

[21] Chapman PB (2011). Improved survival with vemurafenib in melanoma with BRAF V600E mutation. N. Engl. J. Med..

[22] Bucheit AD, Davies MA (2014). Emerging insights into resistance to BRAF inhibitors in melanoma. Biochem. Pharmacol..

[23] Montero-Conde C (2013). Relief of feedback inhibition of HER3 transcription by RAF and MEK inhibitors attenuates their antitumor effects in BRAF -mutant thyroid carcinomas. Cancer Discov..

[24] Calipel A (2006). Extracellular signal-regulated kinase-dependent proliferation is mediated through the protein kinase A/B-Raf pathway in human uveal melanoma cells. J. Biol. Chem..

[25] Yaktapour N (2014). BRAF inhibitor-associated ERK activation drives development of chronic lymphocytic leukemia. J. Clin. Invest..

[26] Poulikakos PI, Zhang C, Bollag G, Shokat KM, Rosen N (2010). RAF inhibitors transactivate RAF dimers and ERK signalling in cells with wild-type BRAF. Nature.

[27] Hatzivassiliou G (2010). RAF inhibitors prime wild-type RAF to activate the MAPK pathway and enhance growth. Nature.

[28] Wrobel CN (2004). Autocrine CSF-1R activation promotes Src-dependent disruption of mammary epithelial architecture. J. Cell. Biol..

[29] Knowlton ML (2010). Profiling Y561-dependent and -independent substrates of CSF-1R in epithelial cells. PLoS One.

[30] Sampaio NG (2011). Phosphorylation of CSF-1R Y721 mediates its association with PI3K to regulate macrophage motility and enhancement of tumor cell invasion. J. Cell. Sci..

[31] Yu R (2018). Inhibition of the CSF-1 receptor sensitizes ovarian cancer cells to cisplatin. Cell. Biochem. Funct..

[32] Sala E (2008). BRAF silencing by short hairpin RNA or chemical blockade by PLX4032 leads to different responses in melanoma and thyroid carcinoma cells. Mol. Cancer Res..

[33] Prahallad A (2012). Unresponsiveness of colon cancer to BRAF(V600E) inhibition through feedback activation of EGFR. Nature.

[34] Orlandi A (2015). BRAF in metastatic colorectal cancer: the future starts now. Pharmacogenomics.

[35] Krittiya K, Kopetz S (2016). BRAF-directed therapy in metastatic colorectal cancer. Cancer J..

[36] Kopetz Scott, McDonough Shannon L, Lenz Heinz-Josef, Magliocco Anthony Martin, Atreya Chloe Evelyn, Diaz Luis A., Allegra Carmen Joseph, Raghav Kanwal Pratap Singh, Morris Van Karlyle, Wang Stephen E., Lieu Christopher Hanyoung, Guthrie Katherine A, Hochster Howard S. (2017). Randomized trial of irinotecan and cetuximab with or without vemurafenib in BRAF-mutant metastatic colorectal cancer (SWOG S1406). Journal of Clinical Oncology.

[37] Desai Jayesh, Markman Ben, Ananda Sumitra, Tebbutt Niall C., Michael Michael, Solomon Benjamin J., McArthur Grant A., Tie Jeanne, Gibbs Peter, Ritchie David, Koldej Rachel, Herschtal Alan, Columbus Ruth, Ashley David M., Lundy Joanne, Kwan Edmond Michael, Waring Paul Michael, Tran Ben (2017). A phase I/II trial of combined BRAF and EGFR inhibition in patients (pts) with BRAF V600E mutated (BRAFm) metastatic colorectal (mCRC): The EViCT (Erlotinib and Vemurafenib in Combination Trial) study. Journal of Clinical Oncology.

[38] Carreno BeatrizM (2009). Immunodeficient mouse strains display marked variability in growth of human melanoma lung metastases. Clin. Cancer Res..

[39] Lee SJ (2015). Natural killer (NK) cells inhibit systemic metastasis of glioblastoma cells and have therapeutic effects against glioblastomas in the brain. BMC Cancer.

[40] Perrotte P (1999). Anti-epidermal growth factor receptor antibody C225 inhibits angiogenesis in human transitional cell carcinoma growing orthotopically in nude mice. Clin. Cancer Res..

[41] Wilson TR (2012). Widespread potential for growth-factor-driven resistance to anticancer kinase inhibitors. Nature.

[42] Abel EV (2013). Melanoma adapts to RAF/MEK inhbitors through FOXD3-mediated upregulation of ERBB3. J. Clin. Invest..

