# Supplementary material for: AZ304, a novel dual BRAF inhibitor, exerts anti-tumour effects in colorectal cancer independently of BRAF genetic status
Source: Br J Cancer. 2018 May 14;118(11):1453–63. doi: 10.1038/s41416-018-0086-x (PMC5988692; doi:10.1038/s41416-018-0086-x)
Supplement: Supplementary file 1 — Supplementary Figure legends [file 41416_2018_86_MOESM1_ESM.docx]

**Supplementary Figure 1 Quantity and histograms analysis of the basal levels of BRAF, p-ERK, p-AKT, p-mTOR, p-SRC, p-STAT3, c-Casps3, c-Casps9 and c-PARP.** The densitometric analysis was performed using the software Image J; Bars are the mean values as mean ± SD. The immunoblots shown in each panel are from three representative experiments. * # P < 0.05, * vs Control, # vs AZ304 respectively.

**Supplementary Figure 2 Weights of xenografts from RKO and Caco-2 cells was measured every 2 days.**
