# Supplementary material for: AZ304, a novel dual BRAF inhibitor, exerts anti-tumour effects in colorectal cancer independently of BRAF genetic status
Source: Br J Cancer. 2018 May 14;118(11):1453–63. doi: 10.1038/s41416-018-0086-x (PMC5988692; doi:10.1038/s41416-018-0086-x)
Supplement: Supplementary file 2 — Supplementary table 1 [file 41416_2018_86_MOESM2_ESM.docx]

| **Supplementary table 1. Cell line background, GI50 and tumor types** | | | | |
| --- | --- | --- | --- | --- |
| **Cell line** | **GI50 (μM)** | **Tumor type** | **BRAF** | **RAS** |
| SK-MEL-24 | 0.08 | Melanoma | V600E | WT |
| Melma-3M | 0.1 | Melanoma | V600E | WT |
| A375 | 0.23 | Melanoma | V600E HM | WT |
| COLO205 | 0.28 | Colon | V600E HT | WT |
| A375pgp | 0.43 | Melanoma | V600E HM | WT |
| SK-MEL-28 | 0.5 | Melanoma | V600E | WT |
| HT29 | 0.51 | Colon | V600E HT | WT |
| SK-MEL-3 | 0.53 | Melanoma | V600E | WT |
| JVM-3 | 1.68 | Lymphoma | K601N | WT |
| A2058 | 3.82 | Melanoma | V600E HT | WT |
| NCI-H1755 | 7.72 | Lung | A468 | WT |
| IM-9 | 0.9 | Lymphoma | WT | NRAS^MT^ |
| MIAPaCa-2 | 1.16 | Pancreas | WT | KRAS^MT^ |
| MDA-MB-231 | 1.99 | Breast | G464V HT | KRAS^MT^ |
| HCT116 | 2.74 | Colon | WT | KRAS^MT^ |
| SW620 | 2.78 | Colon | WT | KRAS^MT^ |
| SK-MEL-2 | 3.91 | Melanoma | WT | NRAS^MT^ |
| NCI-H23 | 3.99 | Lung | WT | KRAS^MT^ |
| RPMI-8226 | 5.37 | Melanoma | WT | KRAS^MT^ |
| PANC-1 | 5.45 | Pancreas | WT | KRAS^MT^ |
| Calu-6 | 5.51 | Lung | WT | KRAS^MT^ |
| L-363 | 7.02 | Leukemia | WT | NRAS^MT^ |
| NCI-H460 | 8.56 | Lung | WT | KRAS^MT^ |
| HCT-15 | 11.5 | Colon | WT | KRAS^MT^ |
| A549 | 16.66 | Lung | WT | KRAS^MT^ |
| SK-MEL-31 | 0.43 | Melanoma | WT | WT |
| LNCaP clone FGC | 3.32 | Prostate | WT | WT |
| SK-OV3 | 6.18 | Ovary | WT | WT |
| PC-3 | 6.68 | Prostate | WT | WT |
| MC-F7/mdr+ | 7.63 | Breast | WT | WT |
| CHL-1 | 8.27 | Melanoma | WT | WT |
| MDA-MB-468 | 8.83 | Breast | WT | WT |
| U937 | 9.37 | Lymphoma | WT | WT |
| MC-F7 | 10.09 | Breast | WT | WT |
| NIH: OVCAR-3 | 10.99 | Ovary | WT | WT |
| SK-BR-3 | 11.61 | Breast | WT | WT |
| DU145 | 11.7 | Prostate | WT | WT |

1. HT = heterozygous; HM = homozygous

**Anti-proliferative activity of AZ304 against a panel of cell lines with different BRAF and RAS genetic status.** Cells were treated with DMSO or multiple concentrations of the compound for 3 days and the cell growth was measured using MTS assay, represented as log mean GI50 (average of three replicates). Cell growth was determined by MTS assay. Percentage of net growth at day 3 (100%) relative to day 0 (0%) was calculated and the concentration of compound required to inhibit growth by 50% determined (GI50). GI50 and the complete list of cell line information all showed above.
