# Supplementary material for: AZ304, a novel dual BRAF inhibitor, exerts anti-tumour effects in colorectal cancer independently of BRAF genetic status
Source: Br J Cancer. 2018 May 14;118(11):1453–63. doi: 10.1038/s41416-018-0086-x (PMC5988692; doi:10.1038/s41416-018-0086-x)
Supplement: Supplementary file 3 — Supplementary table 2 [file 41416_2018_86_MOESM3_ESM.docx]

| **Supplementary table 2** | | **Ki67** | **p-ERK** | **p-EGFR** | **p-AKT** |
| --- | --- | --- | --- | --- | --- |
| **RKO** | **Control** | 11.4 ±1.34 | 10 ±1.87 | 8.2 ±1.30 | 10.8 ±1.64 |
|  | **Cetuximab** | 9 ±2.12 | 6.6 ±1.95 | 4.4 ±1.67 | 5.2 ±1.10 |
|  | **AZ304** | 4.8 ±1.10 | 3.2 ±1.10 | 10 ±1.87 | 4.2 ±2.28 |
|  | **Combination** | 0.8 ±0.84 | 1 ±0.71 | 0.8 ±0.84 | 1.6 ±1.14 |
| **Caco-2** | **Control** | 9.8 ±2.05 | 10.8 ±1.64 | 8.8 ±0.45 | 10.4 ±2.19 |
|  | **Cetuximab** | 6.6 ±1.95 | 3.4 ±0.89 | 3.4 ±0.89 | 3.2 ±0.84 |
|  | **AZ304** | 3.2 ±0.84 | 4.8 ±1.10 | 10.6 ±1.95 | 6.8 ±1.10 |
|  | **Combination** | 0.8 ±1.30 | 1.8 ±1.30 | 1.4 ±1.52 | 1.6 ±1.14 |

Histological score= staining intensity × staining area
