# Supplementary figures and images for: AZ304, a novel dual BRAF inhibitor, exerts anti-tumour effects in colorectal cancer independently of BRAF genetic status
Source: Br J Cancer. 2018 May 14;118(11):1453–63. doi: 10.1038/s41416-018-0086-x (PMC5988692; doi:10.1038/s41416-018-0086-x)

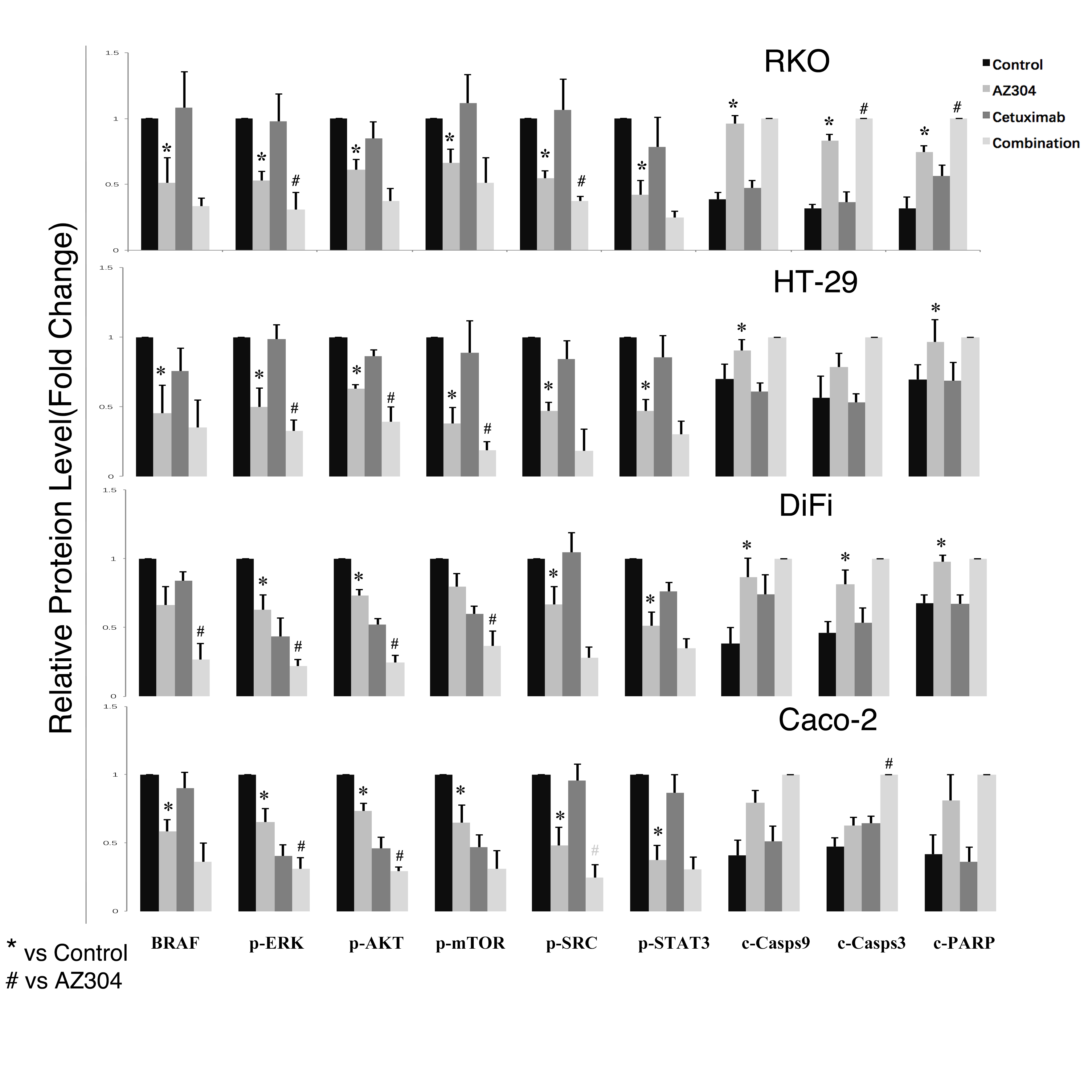

Supplement: Supplementary file 4 — supplymentary fig1 [file 41416_2018_86_MOESM4_ESM.tif]

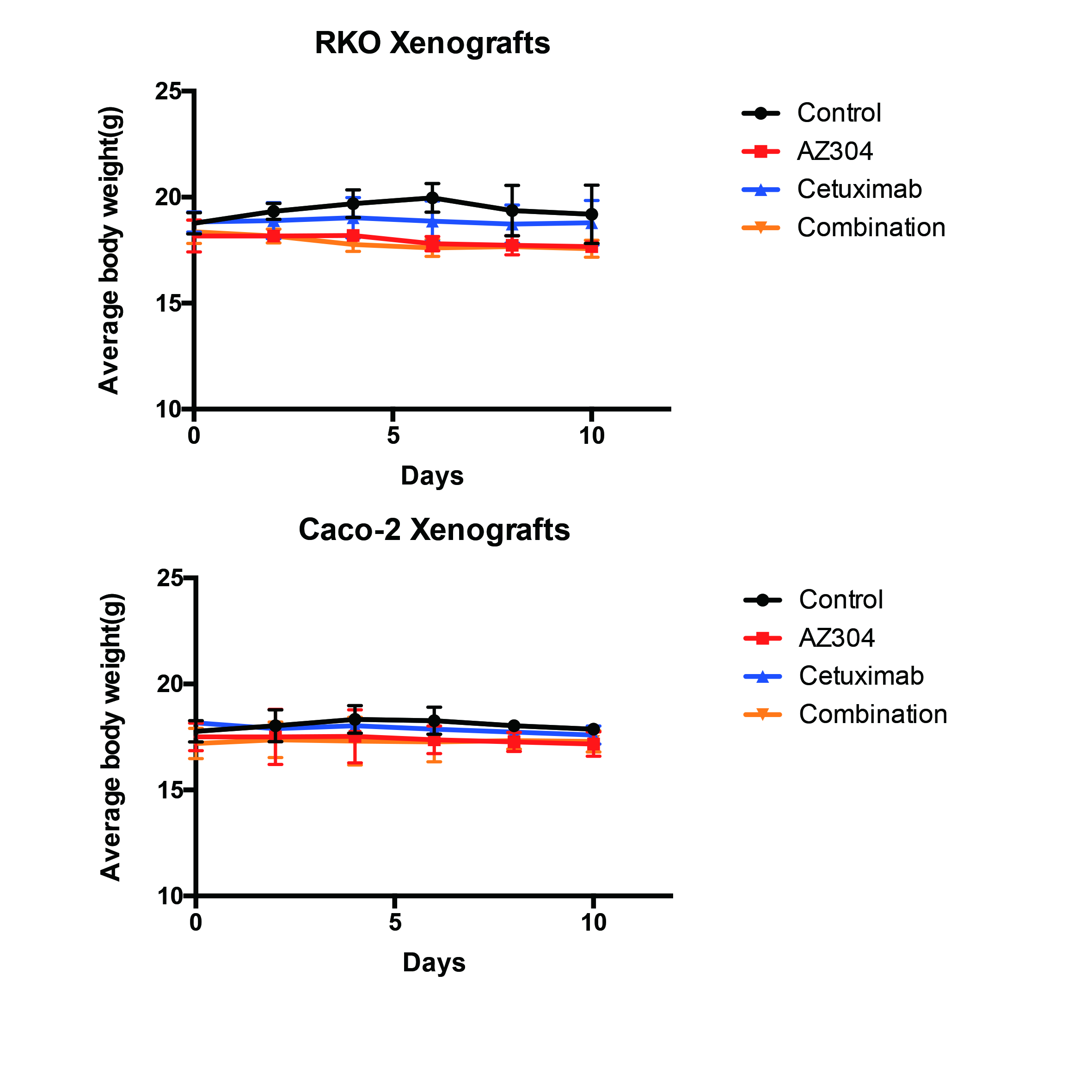

Supplement: Supplementary file 5 — supplymentary fig2 [file 41416_2018_86_MOESM5_ESM.tif]
